# Towards ultrasensitive malaria diagnosis using surface enhanced Raman spectroscopy

**DOI:** 10.1038/srep20177

**Published:** 2016-02-09

**Authors:** Keren Chen, Clement Yuen, Yaw Aniweh, Peter Preiser, Quan Liu

**Affiliations:** 1 School of Chemical and Biomedical Engineering, Nanyang Technological University, Singapore 637457.

## Abstract

We report two methods of surface enhanced Raman spectroscopy (SERS) for hemozoin detection in malaria infected human blood. In the first method, silver nanoparticles were synthesized separately and then mixed with lysed blood; while in the second method, silver nanoparticles were synthesized directly inside the parasites of *Plasmodium falciparum*. It was observed that the first method yields a smaller variation in SERS measurements and stronger correlation between the estimated contribution of hemozoin and the parasitemia level, which is preferred for the quantification of the parasitemia level. In contrast, the second method yields a higher sensitivity to a low parasitemia level thus could be more effective in the early malaria diagnosis to determine whether a given blood sample is positive.

Malaria is a global disease which causes 584,000 deaths per year in the world^1^. Malaria parasites in infected erythrocytes metabolize hemoglobin resulting in the production of hemozoin which can serve as a unique biomarker^2^. Early diagnosis is important in malaria infection management and greatly enhances clinical outcome. Currently microscopic examination of Giemsa-stained blood smears is regarded as the “gold standard” in malaria diagnosis. However, the process is time consuming and requires trained operators to ensure parasite detection especially at low parasitemia levels^3^. Alternative diagnostic techniques that do not require trained personal, are fast and cheap, require minimum sample preparation and are equally sensitive compared to microscopy would greatly aid the global effort of malaria elimination, especially in those regions with low resources.

Numerous approaches have been explored to develop diagnostic tools that are more reliable and/or sensitive than the current gold standard. These include rapid diagnostic tools (RDTs)^4^, cell dyn machine^5^, magneto optical technology (MOT) using polarized light^6^, laser desorption mass spectrometry (LDMS)^7^, magnetic resonance^8^, polymerase chain reaction (PCR)^9^,^10^, quantitative buffy coat method^11^, flow cytometry^12^, serological tests^13^, optical tweezer^14^ and attenuated total reflectance infrared spectroscopy (ATR-IR)^15^. A number of these methods are suitable to be applied in low resource clinical settings but often do not have the desired level of sensitivity needed. These include RDT, CDM, QBC, MOT and a few single cell measurement methods as described below. All of the approaches show reduced sensitivity or alternatively specificity or both particularly at low parasitemia levels. The sensitivity and specificity of RDT can be over 90%, but the accuracy will decrease significantly when the parasite density is lower than 500 parasites/μl^4^ and in addition it is difficult to use RDT to quantify the parasitemia level^4^. The sensitivity values of cell dyn machine (CDM) and quantitative buffy coat (QBC) methods are only 50% (for parasitemia levels less than 0.1%) and 33.3% (for parasitemia levels around 0.02%), respectively. MOT using polarized light requires a strong magnetic field while the sensitivity and specificity are only 26.7% and 80.6%, respectively, in a field test on 46 patient showing uncomplicated malaria infection detected by clinical diagnosis^16^. Flow cytometry can only measure a parasitemia level of 0.1% or higher in culture^12^.

Alternative approaches that can achieve high sensitivity at low parasitemia levels are predominately not suitable for field use due to high cost or low throughput. Laser desorption mass spectrometry is highly sensitive but is expensive and requires extensive sample preparation^7^, while serological tests have high sensitivity and specificity (91% and 86%, respectively) but are time consuming and require expertise in sample interpretation^13^. Similarly approaches using optical tweezers are very time consuming and are therefore not suitable to scan a large volume of samples^14^. Confocal Raman spectroscopy has been conducted to observe hemozoin crystals in the vacuoles of parasites but again its throughput is low^17^. Conventional MRI offers a non-invasive method to demonstrate pathological features of cerebral malaria^18^ but its operation cost is high. To overcome the high cost and bulky equipment limitations of conventional MRI, a micromagnetic resonance relaxometry (MRR) method using a simple low cost and portable MRI device has been reported^19^. MRR can achieve fast and quantitative measurements of the parasitemia level as low as 0.0002% for *P. falciparum* infection but the interference of hemoglobin may affect the accuracy of MRR^19^. PCR is sufficiently sensitive to detect cells infected by a single parasite and track the stage change during parasite development in real time^9^. Importantly it can differentiate between different species of human malaria parasites^20^. However, PCR still has to be seen as time-consuming and sample preparation is still relatively complicated even with the recently developed LAMP based isothermal PCR^21^. ATR-IR has even achieved a sensitivity comparable to PCR^15^. This technique can detect blood infected by parasites in the ring stage with a parasitemia level at 0.00001% and it has been also used to quantify the parasitemia level. However, the actual sensitivity in quantification is only 0.1% and the standard deviation of the estimated parasitemia level is as high as 0.05%. Moreover, it requires an expensive diamond crystal as the sample stage.

Raman spectroscopy has been used to characterize the electronic structure of β-hematin (a biocrystal with close Raman characteristics resemblance to hemozoin) or hemozoin^22^ and its change with drug treatment or disease progression^23^. Moreover, spontaneous Raman spectroscopy combined with other approaches such as confocal microscopy^24^ or optical tweezers^25^ have shown great promise for malaria diagnosis^26^. However, the amount of hemozoin produced in the early ring stages normally seen in peripheral blood is very small and parasites younger than 6 hours post invasion do not contain hemozoin detectable by the dark-field microscopy method^27^, even dark-field microscopy has improved sensitivity as compared to traditional light microscopy^28^. Therefore, the enhancement of Raman signals is crucial for the detection of hemozoin in early ring stages so as to provide the sensitivity needed for diagnosis^17^.

Surface enhanced Raman spectroscopy (SERS) has been reported to enhance the Raman signal of hemozoin or β-hematin by several orders of magnitude^29^. In tip-enhanced Raman spectroscopy (TERS)^30^, an atomic force microscope tip covered with SERS-active silver nanoparticles has been shown to enhance the Raman signal originated from hemozoin in the parasite vacuoles by 10^6^ to 10^7^ folds. The enhancement of Raman signals occurs when hemozoin crystals are in close proximity to or direct contact with noble metal surfaces. Further enhancement can be achieved by surface enhanced Raman resonance scattering (SERRS) when there is close Raman shift match between the excitation laser wavelength and hemozoin electronic transition^31^. This dramatic enhancement of Raman signal suggests the potential use of SERS in early malaria diagnosis where the parasitemia levels and hemozoin concentration in blood samples are very low. However, like other techniques focusing on single cell characterization^17^,^22^,^25^,^32^, the performance of SERS on a single blood cell highly depends on the precision of locating the cell, which can be very challenging at a low parasitemia level. While theoretically possible, to the best of our knowledge SERS has never been used to quantify the parasitemia level of malaria infected blood samples for early malaria diagnosis. Furthermore, the lowest detection limit of SERS in the current literature is about 0.0005%, which was achieved using a gold coated substrate based on butterfly wings^33^. However, this method requires the development of a cost-effective and reproducible method to fabricate complex butterfly wing nanostructures. Moreover, Raman measurements have to be taken from specific locations where the aggregation of hemozoin yields maximum enhancement. This makes it time consuming at a low parasitemia level, since hemozoin spreads sparsely on the substrate when the parasitemia level is low.

We have previously used magnetic field enriched SERS to augment the interaction between β-hematin and silver nanoparticles^31^,^34^ enabling a detection limit as low as 5-nM β-hematin. However, the requirement of an external magnetic field is inconvenient, which could induce additional variation in SERS readings. Here, we report two enhanced SERS methods for the detection of hemozoin from *Plasmodium falciparum* infected human blood. In Method 1, silver nanoparticles are synthesized separately and then mixed with the processed sample just as conducted in other SERS studies^35^,^36^. While in Method 2, silver nanoparticles are synthesized directly inside parasites to achieve closer contact with hemozoin, which is a new technique enabling ultrasensitive detection of hemozoin. Our methods are unique in that it can potentially achieve both cost effectiveness and high sensitivity when combined with the development of a cost effective Raman spectrometer^37^ for spectral measurements and paper based microfluidic chips^38^ for sample preparation. The detection limit achieved by our methods in terms of parasitemia level is comparable to those most sensitive techniques including MRR, PCR and the conventional Giemsa stained microscopy; meanwhile its cost will be only slightly higher than those of RDT.

## Results

**Figure 1:**
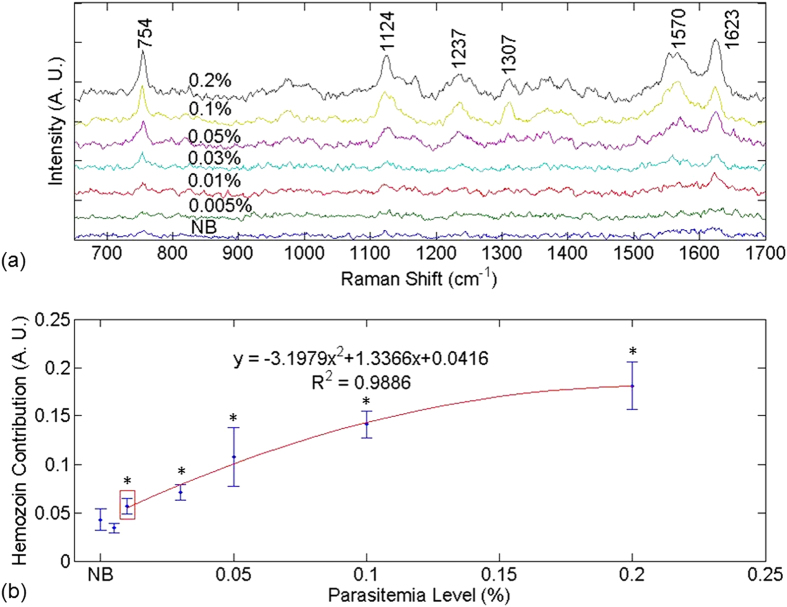
SERS spectra and hemozoin contribution in Method 1. (**a**) SERS spectra contributed by hemozoin in infected blood treated by Method 1. (**b**) Hemozoin contribution as a function of parasitemia level. In (**a**), the data point corresponding to a parasitemia level of 0.01% is marked by a red box. In (**b**), the resulting curve of the second-order polynomial fitting for the data points corresponding to parasitemia levels in the range of 0.01% to 0.2% is shown in red. The data corresponding to normal blood samples, labeled as “NB”, is added manually to facilitate comparison. The y axes in both figures are plotted in arbitrary units, labeled as “A. U.” The asterisks indicate parasitemia levels at which the Raman peak intensity at 1623 cm^−1^ were significantly different from that in the normal blood sample in *t*-test (p < 0.05). The detection limit was determined to be 0.01% parasitemia level (marked by the red box) in this manner.

Figure 1 shows (a) the SERS spectra and (b) hemozoin contribution as a function of parasitemia level for infected and normal blood samples obtained using Method 1. Prominent vibrational features of hemozoin, such as ν_15_ at 754 cm^−1^, ν_2_ at 1570 cm^−1^, and ν_(C=C)_ at 1623 cm^−1^ are shown in most of the spectra in Fig. 1(a). Detailed peak assignments are shown in Supplementary Table 1. Hemozoin contribution refers to the weight of the basic component spectrum corresponding to hemozoin resulting from the least square regression as described in data processing section. The lowest detectable parasitemia level was determined to be 0.01% after conducting a series of *t*-tests to compare the hemozoin contribution in infected blood samples with that in the normal blood sample (p < 0.05). There appears to be a strong correlation between hemozoin contribution and the parasitemia level in Fig. 1(b). The second-order polynomial relation between the SERS intensity and parasitemia level could be explained as follows. Since the volume of silver nanoparticles in this study was fixed, there would be fewer and fewer silver nanoparticles for hemozoin to bind when the concentration of hemozoin increased with the parasitemia level. So the SERS intensity increased linearly with parasitemia level at the beginning but then level off, which looked like a parabola.

**Figure 2:**
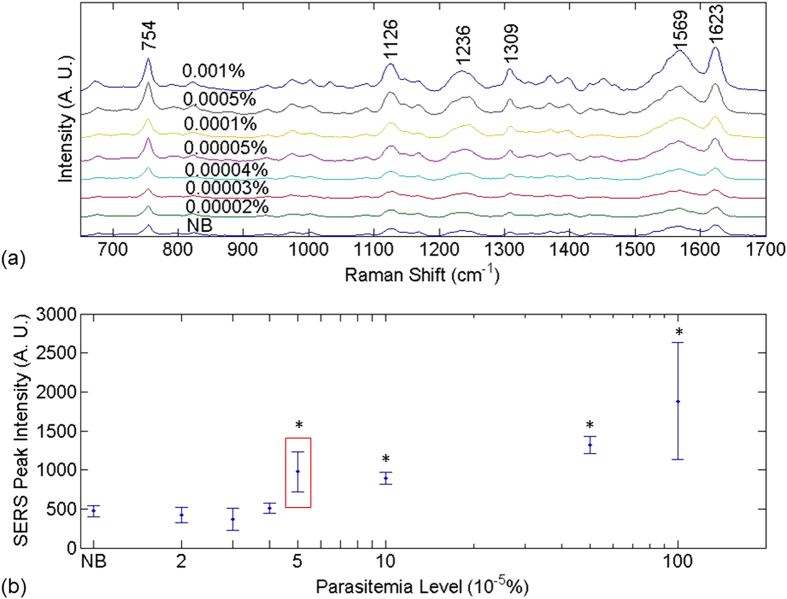
SERS spectra and hemozoin Raman peak distribution in Method 2. (**a**) SERS spectra of normal blood and infected blood sample treated by Method 2. (**b**) SERS peak intensity at 1623 cm^−1^ as a function of parasitemia level. The data corresponding to normal blood samples, labeled as “NB”, is added manually to facilitate comparison. The y axes in both figures are plotted in arbitrary units, labeled as “A. U.” The asterisks indicate parasitemia levels at which the Raman peak intensity at 1623 cm^−1^ were significantly different from that in the normal blood sample in *t*-test (p < 0.001). The detection limit was determined to be 0.00005% parasitemia level (marked by the red box) in this manner.

Figure 2 shows the SERS spectra and ν_10_ peak intensity as a function of parasitemia level obtained using Method 2, in which silver nanoparticles were synthesized inside parasites. Compared with the spectra in Fig. 1, the Raman spectra in Fig. 2 are much stronger. Prominent vibrational features of hemozoin, such as ν_15_ at 754 cm^−1^, ν_2_ at 1570 cm^−1^, and ν_(C=C)_ at 1623 cm^−1^ are shown in all spectra. Detailed peak assignments are shown in Supplementary Table 1. Multiple hemozoin Raman peaks are visible in the spectra of normal blood samples, which can be attributed to hematin converted from hemoglobin during sample preparation. In Method 2, most hemoglobin were filtered out, whereas a tiny amount could still stay in or attached to parasites. During silver nanoparticles synthesis, the use of detergent Triton X-100 and hydroxylamine hydrochloride likely converted the residue hemoglobin to heme and then hematin^39^,^40^ which contributed to the Raman peaks^41^. Note that hematin and hemozoin share most peaks including 1623 cm^−1^. The detection limit for Method 2 is determined to be 0.00005% parasitemia level in the ring stage, after comparing the Raman peak intensity at 1623 cm^−1^ measured from infected blood samples with that from the normal blood sample with *t*-test (p < 0.001). The detection limit is exceptionally low compared to most relevant publications we can find (shown in Table 1).

**Figure 3:**
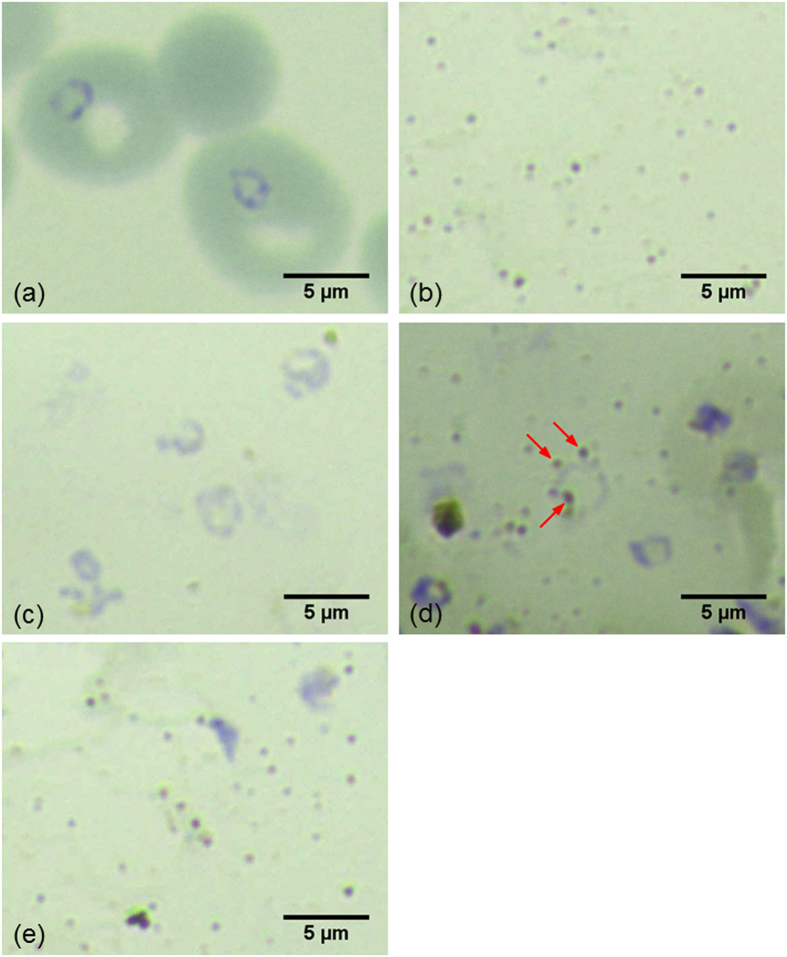
Giemsa stained images of blood samples treated by Method 1 and Method 2. (**a**) a blood sample with parasites in the ring stage prior to cell lysis, (**b**) silver nanoparticles alone, (**c**) a blood sample after blood cell membrane lysis but prior to nanoparticle synthesis treated by Method 2, (**d**) the blood sample after nanoparticle synthesis treated by Method 2 and (**e**) a blood sample treated by Method 1 and then mixed with nanoparticles synthesized separately.

To illustrate the difference in the separation of parasites and nanoparticles between Methods 1 and 2, Fig. 3 shows the Giemsa stained images of a typical infected blood sample, silver nanoparticles and a blood sample treated by Method 2 before and after nanoparticle synthesis as well as a blood sample treated by Method 1 for comparison. Figure 3(a) illustrates the purple rings superimposed on top of red blood cells, which are the stained DNAs of malaria parasites in the ring stage. Figure 3(b) displays the Giemsa stained image of silver nanoparticles serving as a reference to facilitate search for nanoparticles in subsequent images, in which aggregated nanoparticles show up as sparsely distributed brown or black dots. In Fig. 3(c), red blood cells have been lysed as treated by Method 2 so the blood cells do not exist in background, which is visibly different from Fig. 3(a). However, parasites were not lysed because the concentration of the lysing agent was purposefully reduced. For this reason, the stained DNA rings are clearly seen. In Fig 3(d), nanoparticles have been synthesized in the lysed blood sample as treated by Method 2. It can be clearly seen that some nanoparticles are in the close proximity to the DNA rings of parasites as indicated by red arrows. This phenomenon can be observed in roughly one out of every ten rings in samples treated by Method 2. This suggests that silver nanoparticles were more likely to be close to hemozoin inside the same parasites. Because hemozoin was not released to the outside of parasites, the original local hemozoin concentrations in the parasites were maintained, which created higher SERS signals than otherwise. For the purpose of comparison, Fig. 3(e) illustrates the Giemsa stained image of a blood sample treated by Method 1, in which lysed blood samples and parasites were mixed with nanoparticles separately synthesized. Because parasites have been lysed, no DNA rings were observed and hemozoin inside parasites were thus released into the entire sample. Hemozoin concentration in the sample was thus much lower than that in the original parasites. The SERS signal from hemozoin was thus much lower as expected.

## Discussion

Asymptomatic patients with extremely low levels of parasitemia pose a key challenge in any effort to control malaria. The extremely low levels of circulating parasites in the peripheral blood^42^ make accurate diagnosis particularly difficult. Here we present data that clearly demonstrates the feasibility and advantage of surface enhanced Raman spectroscopy for detecting malaria parasites under those circumstances. While the lowest detectable parasitemia level for Method 1 is approximately 0.01% ring stage parasites, comparable to other methods such as QBC and RDTs in malaria diagnosis (Table 1), Method 2 has a detection limit as low as 0.00005%, which equates to approximately 2.5 parasites/μl of blood and is comparable to the most sensitive detection techniques currently available. The strong correlation between hemozoin concentration and parasitemia level seen in Method 1 has the additional advantage of allowing the direct quantification of the parasite level, which would be of great value for the rapid determination of the overall severity of malaria infection. The relatively high ratio of standard deviation to mean seen at each parasitemia level in Method 1 (15% to 58%) is likely the result of variations in contaminating cell debris that adhere to hemozoin crystals and silver nanoparticles during the centrifugation step. This contamination would directly affect the interaction between hemozoin and silver nanoparticles. In addition the lysis method using Triton-X and sonication may not be able to release all hemozoin out of every parasite’s vacuole leading to an overall reduced signal although cost effective. Future work will focus on improving the lysis step to further increase the sensitivity of Method 1.

The exceptionally high sensitivity of Method 2 is better than most existing methods (Table 1). The close proximity of nanoparticles to hemozoin concentrated inside parasites achieved in this approach (Fig. 3) dramatically enhances the Raman signal of hemozoin leading to the improved detection limit. While very promising, one limitation of synthesizing nanoparticles inside parasites is the inconsistent distribution of accumulated hemozoin within the parasites. The Raman signal acquired from a location with parasites and nanoparticles inside would be much stronger than that from another location without them. Because the distribution of parasites with nanoparticles inside is discrete and usually inhomogeneous in the sample, the variation in Raman peak intensities from random locations or different batches is higher than that in Method 1. For the same reason, there is no strong correlation between the SERS peak intensity and the parasitemia level. However, with the lowest detectable parasitemia level at 0.00005%, Method 2 can be utilized to determine whether a patient is infection positive or to screen high-risk population.

The simplicity and speed of our SERS based techniques indicate their great promise in malaria diagnosis. In the case of *P. falciparum* infections in which parasites observed in peripheral blood are predominantly in the ring stage, Method 1 would allow the relatively accurate determination of the parasitemia level. While infections containing asynchronous stages would be more difficult to accurately quantify as the hemozoin level varies between the different stages unless an extra synchronization step is employed.

One possible challenge for SERS based techniques that rely on solely hemozoin measurements is its inability to distinguish between hemozoin produced by a viable parasite and that still circulating in blood due to a prior infection. One earlier work has shown that detection of residual hemozoin using a Cell-Dyn automated haematology analyser from a prior malaria infection can be achieved up to 4 weeks after treatment^5^. This suggests the more sensitive SERS technique would also detect hemozoin from past malaria infections, which will somewhat limit the ability of this approach to making a definitive diagnosis of an active malaria infection. However the technique can be used to accurately exclude a malaria infection and provide crucial data of either a recent or current asymptomatic infection. This information would be of immense value in the later stages of a malaria eradication campaign^43^,^44^.

Compared to the previously published SERS method^33^, our strategy provides the added benefit of easier nanoparticle preparation, lower cost and higher sensitivity. Moreover, the SERS measurements in our methods were performed on random locations rather than on selected hot spots resulting in increased speed. Although an automated method for hot spot searching has been developed^45^,^46^, such a method requires scanning the entire sample and its accuracy can be affected by fluorescence, which makes it time-consuming and inconvenient. The waiver of hot spot search in our techniques would facilitate the development of fast and convenient automated SERS measurements for field use in the future. In addition, Method 1 also provides the possibility to quantify parasitemia levels directly, which was not available in the published SERS method^33^ possibly due to large variation in SERS peak intensities. While the greater variation of spectral intensities in Method 2 is one limitation, two potential strategies could be used to minimize it. One strategy is to combine Method 2 with an automated hot-spot searching method. With a proper threshold setting, one can scan the entire sample to measure only “hot spots” in the sample to minimize variation. The other potential way is to concentrate the parasites with nanoparticles using an isolating device such as MidiMACS (Miltennyi Biotec, Bergisch Gladbach, Germany), to minimize the chance of measuring regions that contain no parasites or nanoparticles.

The improvements described here now make SERS comparable to the standard microscopy method in terms of detection limit but without the need of an experienced operator. Most steps in the two methods reported in this study could be implemented in a microfluidic chip to enable automatic sample preparation. A paper based microfluidic chip^38^ could further lower the cost of sample preparation. The bottleneck in the high cost of a Raman spectrometer with decent spectral resolution could be overcome by a much cheaper alternative method, in which a cost effective and compact Raman spectrometer^37^ acquires raw Raman spectra with low spectral resolution and a spectral recovery method we developed recently^47^ is used to reconstruct the Raman spectra with high spectral resolution. Therefore the two SERS based methods developed in this study in combination with the cost effective Raman spectrometer we are currently developing hold great potential for malaria diagnosis even in low-resource regions.

## Methodology

### Parasite culture

Plasmodium falciparum parasite (3D7) were cultured in fresh red blood cells (RBCs) at 5% hematocrit with media constituted of bicarbonate buffered RPMI 1640 (RPMI 1640 Medium, Life Technologies, Grand Island, USA) supplemented with 5% albumax (AlbuMAX^®^ I Lipid-Rich BSA, Life Technologies, Grand Island, USA), 200 μM hypoxanthine and 20 μg/ml gentamycin as described previously^48^. The cells were maintained at 37 °C and a gas mixture of 5% CO_2_, 1% O_2_ and 94% N_2_. After 5 cycles of parasite growth, the mixed culture was synchronized using 5% D-sorbitol (D-Sorbitol, Sigma, St. Louis, USA) treatment^49^ and allowed to grow one more cycle then were washed to get rid of floating hemozoin. Parasites were mounted onto glass slides and stained with Giemsa (Giemsa stain, Sigma, St. Louis, USA). The parasitemia level was estimated by manual counting under microscopic observation after Giemsa staining and confirmed by staining cells with syber-green (SYBER^®^Green, Life Technologies, Grand Island, USA) dye and counting with BD LSRII FACS (BD Biosciences, San Jose, USA). Appropriate dilutions were made using non-infected blood in RPMI 140 for further analysis. Optical microscopy was used to confirm the stages of parasites. It turned out that nearly 98% of parasites were in the ring stage and 2% in the early trophozoite stage in this study.

### Sample preparation in Method 1 (synthesizing nanoparticles separately)

In Method 1, silver nanoparticles were synthesized separately and then mixed with isolated hemozoin. Silver nanoparticles were synthesized using the reduction method^31^,^34^,^50^. A total of 33-μl Triton X-100 (Triton X-100 Detergent, Bio-Rad Laboratories, Hercules, USA) was mixed with 5-ml hydroxylamine hydrochloride (0.03 mM, MP Biomedicals, Santa Ana, USA) and NaOH (0.15 mM). The mixture was added drop-wise over a period of 8 min to 45 ml aqueous AgNO_3_ (Silver nitrate, Merck, Kenilworth, USA) with a concentration of 1.11 mM. The resulting solution was then sonicated (Elma E30H, Elma, Wetzikon, Switzerland) for 30 min. Finally, the generated silver nanoparticles were centrifuged and resuspended in 5 ml solution. The suspension was sonicated for 5 min for later use.

Hemozoin crystals were extracted by simple cell lysis and centrifuging. A total of 10 μl infected blood sample were dispersed in 50 ml deionized water mixed with 100 μl Triton X-100 and then sonicated for 5 min. Released hemozoin crystals were collected by centrifuging at 5000 rpm for 5 min (Sartorius 2-16, Sigma Laborzentrifugen, Ostrode, Germany) and resuspended in 5-ml NaOH solution (0.05 mM).

Then silver nanoparticles suspension and hemozoin suspension were mixed together (1:1 v/v). The mixture was sonicated for another 2 min. During Raman measurements, the sample was smeared on a slide covered by aluminum foil as the substrate. On each slide, five random locations were selected for Raman measurements while the sample was still wet.

### Sample preparation in Method 2 (synthesizing nanoparticles inside parasites)

In Method 2, silver nanoparticles were synthesized inside parasites directly to achieve closer contact with hemozoin than Method 1. It should be highlighted that this method keeps the local hemozoin concentration in parasites because it does not break down parasites. A total of 10-μl lysed blood was dispersed in 50-ml deionized water and then sonicated for 5 min to achieve blood cell lysis. Different from the cell lysis procedure in Method 1, Triton X-100 was removed in the lysis step of Method 2 to prevent the lysis of parasites. The mixture was pushed through a filter with a pore size of 0.2 μm (Supor Syringe filters, Pall Life Science, Washington, USA) in a syringe to remove hemoglobin in the lysed blood sample. Then, the residue in the filter was flushed out and suspended in 45-ml AgNO_3_ (Silver nitrate, Merck, Kenilworth, USA) solution with a concentration of 1.11 mM. Then 33-μl Triton X-100 (Triton X-100 Detergent, Bio-Rad Laboratories, Hercules, USA) was mixed with 5-ml solution of hydroxylamine hydrochloride (0.03 mM, MP Biomedicals, Santa Ana, USA) and NaOH (0.15 mM), which was then added drop-wise over a period of 8 min to aqueous AgNO_3_ to reduce silver. In this step, the volume of Triton X-100 was low and it mostly bonded to silver nanoparticles to prevent them from aggregation, which limited its effect on cell lysis to a low level. The resulting solution was then sonicated (Elma E30H, Elma, Wetzikon, Switzerland) for 30 min. Finally, the sonicated solution was pushed through a 0.2-μm filter (Supor Syringe filters, 25 mm, 0.2 μm, Pall Life Science, Washington, USA) in a syringe to filter away small excessive nanoparticles and chemicals and leave analyte on the filter paper. The filter paper was taken out and deposited on a slide covered by aluminum foil for Raman measurements. For each filter paper, five random locations were selected for Raman measurements.

### Raman measurements and data processing

A micro-Raman spectrometer system (inVia, Renishaw, Aberdeen, UK) coupled with a microscope (Alpha 300, WITec, Ulm, Germany) in a backscattering geometry was used to measure all Raman spectra. The system was coupled to a Czerny-Turner type spectrograph (f = 250 mm) with a holographic grating (1800 grooves/mm) and a RemCam CCD detector (inVia, Renishaw, Aberdeen, UK), which yields a spectral resolution of 2 cm^−1^. A 633-nm diode laser (Renishaw, Aberdeen, UK) was used for excitation with a focal spot size about 3 μm on the sample through a microscope objective lens (5X, NA = 0.2, Leica, Wetzlar, Germany). The exposure time was 10 seconds and the excitation was 2.5 mW for all SERS measurements. The combination of similar exposure time and excitation power was also used previously in our magnetic enriched SERS study^31^, which can achieve the tradeoff between the maximization of SERS intensity and minimization of the thermal effect.

In each sample, the Raman spectra were measured from five random locations then averaged to reduce variation. No hot spot search was done during spectral measurements in Method 1 or Method 2. The measurement at each parasitemia level was repeated in seven different samples in Method 1 and five different samples in Method 2 for every concentration to calculate the standard deviation. In each raw spectrum, a fifth-order polynomial was used to fit the fluorescence background and then the polynomial was subtracted from the raw spectrum to generate the clean Raman spectrum.

To obtain the contribution of hemozoin alone in the measurement of an infected blood sample for convenient visualization in Method 1, the clean Raman spectrum of an infected blood sample was modeled as the summation of the contribution from the normal blood sample and that from hemozoin. The Raman spectra of the latter two basic components were measured separately (see Fig. 1 in Supplementary Information), in which the basic component spectrum of hemozoin was replaced by that measured from β-hematin at a concentration 10 μg/ml. This is justified by the fact that β-hematin is equivalent to hemozoin in terms of Raman features^29^,^41^ and much easier to synthesize. Then the contribution of each component to the infected blood spectrum was estimated using a least square regression method^51^ implemented by the lsqcurvefit function in MATLAB with an option of trust-region-reflective algorithm.

### Detection limit estimation

To determine the detection limit for Method 1, a *t*-test was conducted to evaluate the difference in hemozoin contribution between every infected blood sample with a progressively decreasing parasitemia level and the normal blood sample. When there was no difference between the two samples, the parasitemia level in the previous infected blood sample that still demonstrated a significant difference in hemozoin contribution compared to the normal blood sample was determined to be the smallest detectable parasitemia level. The lowest detectable parasitemia level for Method 2 was determined in the same manner by conducting a series of *t*-tests except that the Raman peak intensity at 1623 cm^−1^ instead of hemozoin contribution was compared.

### Ethics statement

The use of human blood strictly followed protocols and guidelines that were approved by the domain-specific review board of Nanyang Technological University (IRB number: NTU-IRB 11/12/2011). Blood component collection service was provided by Blood Transfusion Service and Blood Donation Centre of National University Hospital. All individuals gave informed consent.

## Additional Information

**How to cite this article**: Chen, K. *et al.* Towards ultrasensitive malaria diagnosis using surface enhanced Raman spectroscopy. *Sci. Rep.* **6**, 20177; doi: 10.1038/srep20177 (2016).

## Supplementary Material

Supplementary Information

## Figures and Tables

**Table 1:** Sensitivities of current methods in malaria diagnosis.

Methods	Detection limit in the ring stage	Original statement without conversion
Giemsa stained microscopy	4 to 20 parasites/μl in thick smear	4 to 20 parasites/μl in thick smear for all different stages. The threshold may be higher in the field for the early stage^4^,^5^.
Flow cytometry	100000 parasites/μl	This technique is useful for a parasitemia level around 2% and maybe more helpful in a late stage^12^.
Magneto optical technology (MOT) using polarized light	10000 parasites/μl*	0.002% parasitemia level in the trophozoite stage^6^.
Quantitative Buffy Coat (QBC) Test	1000 parasites/μl	The sensitivity will be lower than 30% when the parasitemia level is lower than 1000 parasites/μl^11^.
Rapid diagnostic tools (RDTs)	100 to 500 parasites/μl for *P. falciparum* infection	100 to 500 parasites/μl for *P. falciparum* infection in all different stages^4^,^52^.
Cell dyn machine	27.786 parasites/μl (in unknown stages)	The parasitemia level of clinical samples varies from 320 to 285714 parasites/ml with an average of 27786 parasites/ml, using Cell dyn 3700^5^.
Laser desorption mass spectrometry (LDMS)	10 parasites/μl	10 parasites/μl in the ring stage^7^.
Micromagnetic Resonance Relaxometry (MRR)	10 parasites/μl for *P. falciparum* infection	0.0002% parasitemia level for *P. falciparum* infection mostly in the ring stage and 0.0001% for *P. berghei* mouse model^19^.
Polymerase Chain Reaction (PCR)	around 0.7 parasites/μl for *P. falciparum* infection	Real-time PCR assay shows a detection limit of 0.7, 4, and 1.5 parasites/μl for *P. falciparum*, *P. vivax*, and *P. ovale*, respectively in different stages^9^,^10^.
Attenuated total reflectance infrared spectroscopy (ATR-IR)	0.5 parasites/μl	The absolute detection limit was found to be 0.00001% parasitemia in the ring stage^15^.
Surface enhanced Raman spectroscopy (SERS) using butterfly-wing nanostructures	25 parasites/μl	0.0005% parasitemia level in the ring stage^33^.
Method 1 in our study	500 parasites/μl	0.01% parasitemia level in the ring stage.
Method 2 in our study	2.5 parasites/μl	0.00005% parasitemia level in the ring stage.

The percent parasitemia level is converted to the number of parasites per microliter with the assumption that normal blood in human body contains 5 × 10^9^ RBCs/ml.

^*^The detection limit of the MOT method was converted from the hemozoin concentration^6^ to the parasitemia level in the original paper. Their assumptions in conversion included: A) hemoglobin concentration in a healthy human is around 340 g/l; B) parasites in the trophozoite stage convert 50% of hemoglobin to hemozoin and then yield around 0.6 pg hemozoin per cell. The detection limit of 0.06 μg/ml in hemozoin concentration was converted to a parasitemia level of 100 parasites/μl in the trophozoite stage^6^. For consistency with all other techniques, the parasitemia level in the trophozoite stage is converted to that in the ring stage here for the same hemozoin concentration based on the assumption that hemozoin concentration in the trophozoite stage is 100 times that in the ring stage^53^,^54^. This yields a sensitivity of 10000 parasites/μl in the ring stage for MOT.
