# Corrigendum: Towards ultrasensitive malaria diagnosis using surface enhanced Raman spectroscopy

**DOI:** 10.1038/srep44511

**Published:** 2017-03-23

**Authors:** Keren Chen, Clement Yuen, Yaw Aniweh, Peter Preiser, Quan Liu

Scientific Reports 6: Article number: 2017710.1038/srep20177; published online: 02 09 2016; updated: 03 23 2017

In this Article, Yaw Aniweh and Peter Preiser are incorrectly listed as being affiliated with the ‘School of Chemical and Biomedical Engineering, Nanyang Technological University, Singapore 637457’. The correct affiliation for both is listed below:

‘School of Biological Sciences, Nanyang Technological University, 637551, Singapore’.

