# Supplementary material for: Towards ultrasensitive malaria diagnosis using surface enhanced Raman spectroscopy
Source: Sci Rep. 2016 Feb 9;6:20177. doi: 10.1038/srep20177 (PMC4746575; doi:10.1038/srep20177)
Supplement: Supplementary Information [file srep20177-s1.doc]

**Towards ultrasensitive malaria diagnosis using surface enhanced Raman spectroscopy**

**Keren Chen, Clement Yuen, Aniweh Yaw, Peter Preiser, Quan Liu***

**School of Chemical and Biomedical Engineering, Nanyang Technological University, Singapore 637457**

**quanliu@ntu.edu.sg**

**Supplementary Materials**


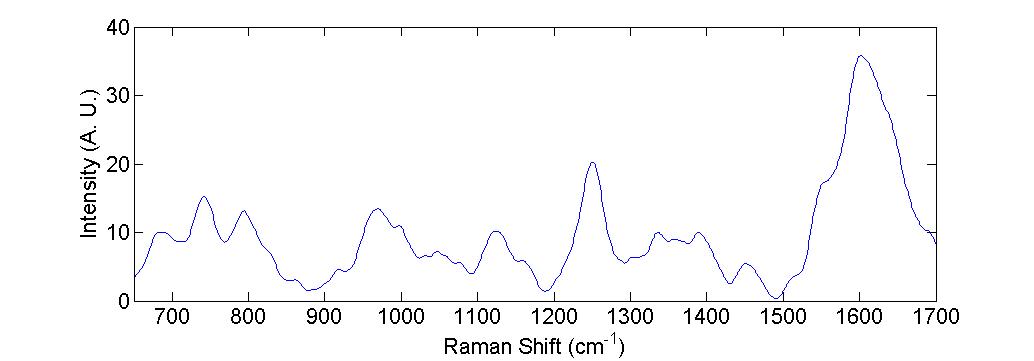


(a)


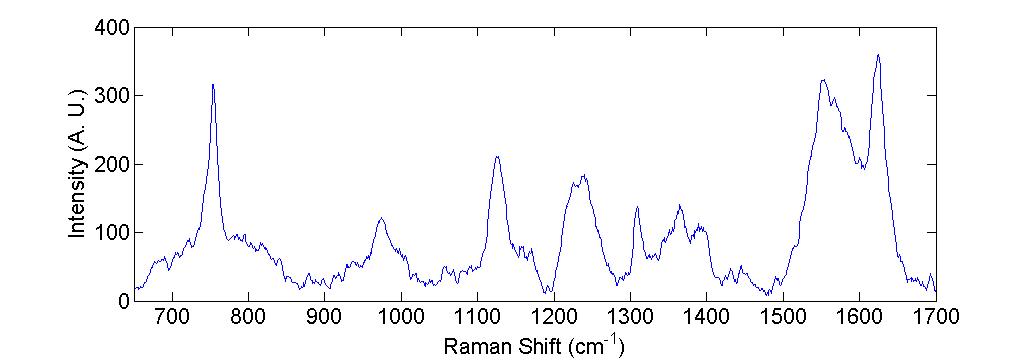


(b)

Supplementary Fig. 1(a) Averaged spectrum of normal blood samples in Method 1. (b) Typical spectrum of β-hematin samples at a concentration of 10 μg/ml.

In the average spectrum of normal blood samples shown in Supplementary Fig. 1(a), there is no peak at 1623 cm^-1^. Supplementary Fig. 1(b) demonstrates the typical spectrum of β-hematin, which is equivalent to hemozoin in Raman features[^55^](#_ENREF_55), with a prominent peak at 1623 cm^-1^. These two spectra were used as two basic biochemical components in a method of least square regression[^51^](#_ENREF_51) to calculate the contribution of hemozoin to the Raman spectrum of an infected blood sample with an assumption that the Raman spectrum of infected blood can be modeled as the linear summation of those contributed by normal blood and hemozoin separately. Fig. 1 in the text shows the contribution of hemozoin as a function of parasitemia level obtained in this manner.


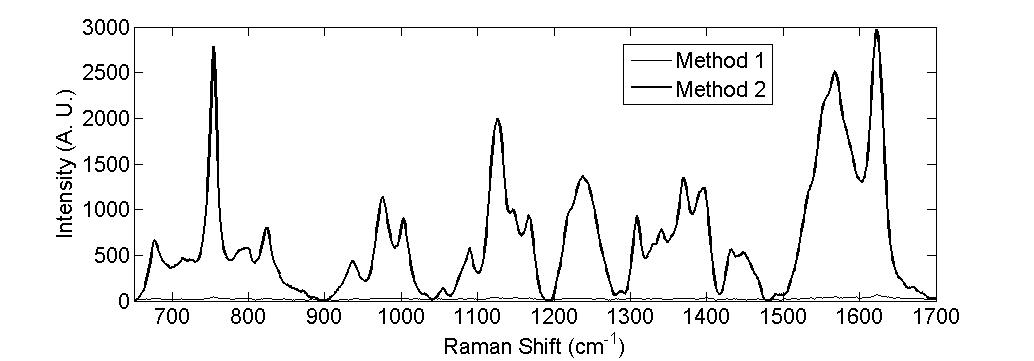


Supplementary Fig. 2 Comparison in spectral magnitude between Method 1 and Method 2. The spectra were acquired from infected blood samples both at a parasitemia level of 0.01%.


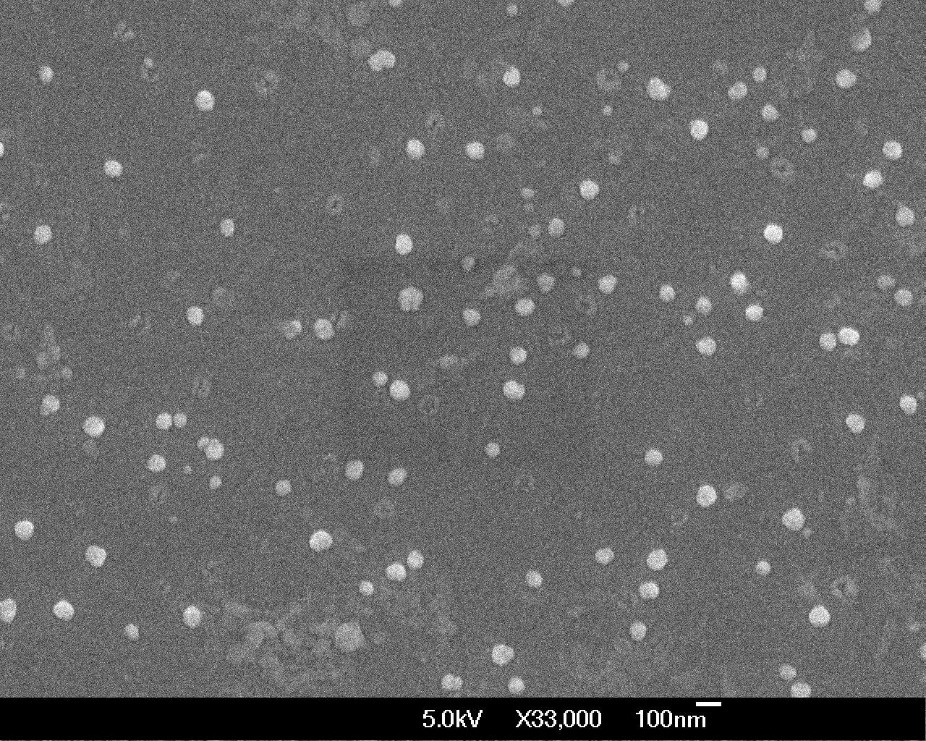


Supplementary Fig. 3 FESEM image of silver nanoparticles synthesized in Method 1. The nanoparticle size ranges from 50 to 100 nm.

Raman peaks of spectra acquired in Method 1 and Method 2 are compared in Supplementary Table 1. It shows that two sets of spectra share most Raman peaks including those prominent ones including ν_15_ at 754 cm^-1^, ν_22_ at 1122 cm^-1^, ν_21_ at 1307 cm^-1^, ν_2_ at 1570 cm^-1^ and ν_(C=C)_ at 1623 cm^-1^. The locations of these peaks are similar to those Raman peaks reported for hemozoin in parasites’ vacuoles[^17^](#_ENREF_17) that are also listed for comparison. The small differences in peak wavenumbers among these methods could be attributed to the variation from SERS measurements in our methods to spontaneous Raman measurements in the literature and the limited spectral resolution of Raman instrumentation. The differences between Method 1 and Method 2 is that ν_41_ at 1337 cm^-1^ showing up in Method 1 is labeled as “absent” in Method 2. The inspection of raw spectra for Method 2 shows that, the peak at 1337 cm^-1^ actually shows up in 2 out of 5 raw spectra in Method 2, although not significant. But the averaging step flattens the peak region. This peak can be assigned to residual DNA[^56^](#_ENREF_56) from white blood cells and/or parasites that were not completely removed during blood sample preparation. The peaks at 1054 cm^-1^, 1033 cm^-1^, 714 cm^-1^ and 676 cm^-1^ are shown in our results but not in the literature, which can be all assigned to proteins in red blood cells[^57-59^](#_ENREF_57).

Supplementary Table 1. Comparison in the peaks of SERS spectra between Method 1 and Method 2. The peaks of spontaneous Raman spectra measured from hemozoin in parasites’ vacuoles in the literature[^17^](#_ENREF_17) and their assignment are also listed for comparison[^60^](#_ENREF_60).

| Wavenumber in Method 1 (cm^-1^) | Wavenumber in Method 2 (cm^-1^) | Wavenumber in the literature (cm^-1^)[^17^](#_ENREF_17) | Assignment[^60^](#_ENREF_60) |
| --- | --- | --- | --- |
| 1623 | 1623 | 1627 | ν_(C=C)_ |
| 1570 | 1569 | 1569 | ν_2_ |
| 1432 | 1431 | 1430 | ν_28_ |
| 1399 | 1392 | 1398 | ν_20_ |
| 1371 | 1372 | 1375 | ν_4_ |
| 1337 | Absent | 1339 | ν_41_ |
| 1307 | 1309 | 1308 | ν_21_ |
| 1237 | 1236 | 1239 | Not available |
| 1169 | 1168 | 1220 | ν_30_ |
| 1146 | 1146 | 1147 | Not available |
| 1124 | 1126 | 1122 | ν_22_ |
| 1091 | 1091 | 1091 | ν_23_ |
| 1053 | 1054 | Absent | Not available |
| 1033 | 1033 | Absent | Not available |
| 1003 | 1002 | 1003 | ν_47_ |
| 976 | 974 | 973 | ν_46_ |
| 938 | 935 | 942 | Not available |
| 825 | 823 | 821 | Not available |
| 793 | 789 | 797 | ν_6_ |
| 754 | 754 | 754 | ν_15_ |
| 714 | 714 | Absent | Not available |
| 676 | 677 | Absent | ν_7_ |

**References**

55 Bohle, D. S. *et al.* in *Inorganic and Organometallic Polymers II* Vol. 572 *ACS Symposium Series* Ch. 37, 497-515 (American Chemical Society, 1994).

56 Ichimura, T., Hayazawa, N., Hashimoto, M., Inouye, Y. & Kawata, S. Tip-Enhanced Coherent Anti-Stokes Raman Scattering for Vibrational Nanoimaging. *Physical Review Letters* **92**, 220801 (2004).

57 Silva, B. L. *et al.* Polarized Raman Spectra and Infrared Analysis of Vibrational Modes in L-Threonine Crystals. *Brazilian Journal of Physics* **28**, 19-24 (1998).

58 Chen, Y. *et al.* Raman Spectroscopy Analysis of the Biochemical Characteristics of Molecules Associated with the Malignant Transformation of Gastric Mucosa. *PLoS ONE* **9**, e93906, doi:10.1371/journal.pone.0093906 (2014).

59 Sijtsema, N. M., Otto, C., Segers-Nolten, G. M. J., Verhoeven, A. J. & Greve, J. Resonance Raman Microspectroscopy of Myeloperoxidase and Cytochrome b558 in Human Neutrophilic Granulocytes. *Biophysical Journal* **74**, 3250-3255, doi:10.1016/S0006-3495(98)78031-2 (1998).

60 D Carter III, W. *Raman spectroscopic study of single red blood cells infected by the malaria parasite Plasmodium falciparum*, Master's thesis, University of Central Florida Orlando, Florida, (2007).
